# Dose variations for biopsy, puncture and drainage under CT guidance: A national survey in 1709 patients

**DOI:** 10.1016/j.redii.2023.100025

**Published:** 2023-03-01

**Authors:** Laure Berny, Joël Greffier, Chris Serrand, Djamel Dabli, Fabien De Oliveira, Hélène de Forges, Jean-Paul Beregi, Julien Frandon

**Affiliations:** a Department of medical imaging, CHU de Nîmes, université de Montpellier, Medical Imaging Group Nîmes, place du Pr.-Robert-Debré, 30029 Nîmes, France; b Department of biostatistics, clinical epidemiology, public health, and innovation in methodology (BESPIM), CHU de Nîmes, place du Pr.-Robert-Debré, 30029 Nîmes, France

**Keywords:** Physics, Radiology, interventional, Radiation exposure, Multidetector computed tomography

## Abstract

**Background:**

A nation-wide study recently published the dose reference levels for the main CT-guided interventional procedures in 5001 patients. We assessed the impact of patient's age, sex and targeted organ on the patient dose during thoracic and abdominopelvic biopsies and punctures/drainages.

**Patients and methods:**

Data were extracted from the previous nationwide study. All biopsies, punctures and drainages for thoracic or abdominopelvic locations performed between January 2017 and June 2019 in all participating centers were included in the study. Multivariable analyses were carried out using a linear regression of the dose-length product (DLP) log, adjusted to age, sex, anatomical location, number of helical acquisitions and inclusion center.

**Results:**

Of the 5001 patients of the initial study, 2383 benefited from thoracic or abdominopelvic procedures, including 674 percutaneous destructions excluded. 1709 patients (44 centers), 1045 men, 664 women, median age 64.4 ± 14.0 years were included. The mean DLP was 751.2 ± 642.7mGy.cm. It was significantly higher in men than women (*p* = 0.0005) and higher for abdominopelvic procedures than for thoracic locations (*p*<0.0001).

**Conclusion:**

Doses delivered to patients for abdominal and thoracic biopsies and punctures/drainages performed under CT guidance depend on gender and location. Furthers studies taking into account the patient's morphology and anatomical location of the procedure would allow proposing finer dose reference levels.

## Highlights

• Doses delivered to patients (DLP) during CT-guided biopsies, punctures/drainages remain high.
• Mean DLP was higher in men vs women and in abdominopelvic procedures vs thoracic.
• Studies including patient's morphology should propose finer dose reference levels.

## 1 Introduction

CT scan is, to date, one of the main methods of guidance in interventional radiology [1], [2], [3], combining high spatial resolution, high contrast resolution and wide-field analysis. Thoracic and abdominal biopsies, drainages and punctures are among procedures usually performed under CT-guidance. It allows for these procedures a good control of the needle progression and of the structures crossed during the procedure. However, the doses delivered to the patients are often high [1], [2], [3]. Dose optimization is thus essential and constitute a challenge. To optimize the dose, studies on technical changes in CT acquisitions and reconstruction parameters have been published [4,5]. The radiologic interventional practice could also be modified to reduce the dose delivered by minimizing preliminary planning imaging [6], decreasing the size and number of helicoidal acquisitions [7], and prefer sequential mode when possible [7,8].

To optimize doses, comparison with reference levels must be performed. Recently, a multicenter nation-wide study has proposed the national reference levels for the main interventional procedures performed under CT guidance on 5001 patients from 49 centers [9]. The doses delivered showed great variability for the same category of procedures, suggesting demographical and morphological factors may have an impact on the dose. Morphological factor such as body mass index (BMI) were not recorded. Although demographical data were recorded, the impact of clinical parameters such as patient age and sex or anatomical location of CT scan on the dose delivered to the patient was not studied.

The purpose of the present study was to assess the influence of the patient's age, sex and targeted organ on the dose delivered to the patient during CT-guided thoracic or abdominal biopsies, punctures and drainages.

## 2 Patients and methods

### 2.1 Data extraction

Based on the data from the previous national study [9], we extracted between June and August 2020 all biopsies, punctures and drainages for thoracic or abdominopelvic locations performed between January 2017 and June 2019 in all participating centers. Methods of study design and data collection of the original study, performed between January and September 2019, were previously published [9]. The study was approved by the local Institutional Review Board (BLINDED).

In the present study, we extracted from the original database, the total dose-length product (DLP), anatomical location of the treated organ and patient's age and sex. The patient's age was categorized in 3 classes corresponding to the three tiertiles, < 60 years old, between 60 and 70, and ≥ 71 years old. Regarding the targeted organ location, they were divided into 9 locations: lung, kidney, retroperitoneum, pelvis, liver, pancreas, gall bladder, adrenal and “other abdominal locations”. The centers were asked to provide the number of their helical acquisitions corresponding to full CT scan of the targeted organ location. Other acquisitions used during guidance such as helical, sequential, fluoroscopic modes were not reported.

### 2.2 Statistical analysis

As the DLP variable does not follow a Gaussian distribution, a logarithmic transformation was performed. Multivariable analysis were carried out using a linear regression of the DLP log. Multivariable analysis were adjusted according to age, sex, anatomical location, number of helical acquisitions and inclusion center. The DLP means are presented with their 95% confidence interval (95% CI) and their corresponding *p*-values after exponential transformation of the linear regression estimates. Statistical analysis was performed using SAS 9.4® 2017 software (SAS Institute Inc, Cary, NC, USA).

## 3 Results

### 3.1 Patients

Among the 5001 patients from 51 centers included in the initial study, 2383 patients benefited from thoracic or abdominopelvic procedures, including 674 patients who underwent percutaneous destructions, excluded from the present analysis. A total of 1709 patients from 44 centers (1045 men, 664 women) underwent a biopsy, puncture or drainage in thoracic or abdominopelvic locations and were included in the analysis. Biopsies were performed in 1248 patients, 649 were thoracic and 599 abdominopelvic biopsies. Regarding the 461 punctures and drainages, 111 patients underwent thoracic punctures/drainages and 350 patients abdominopelvic punctures or drainages. In the overall population, the mean age was 64.4 ± 14.0 years and the mean DLP was 751.2 ± 642.7mGy.cm.

### 3.2 Dose multivariable analysis

**Fig. 1:**
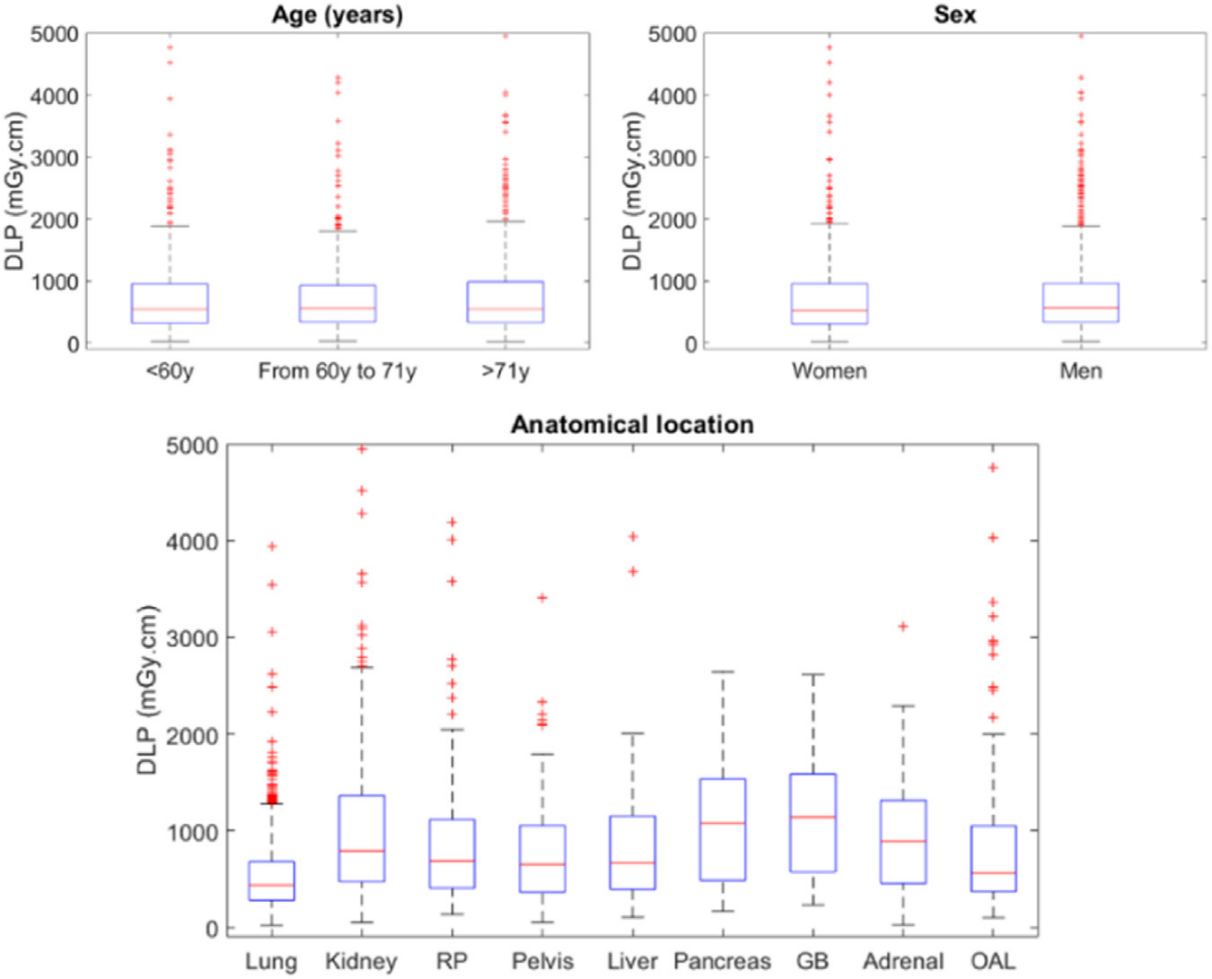
Boxplot of total DLP (*n* = 1709 patients) as function of patient's age, sex, and anatomical location of the organ treated during biopsy, puncture or drainage at thoracic and abdominopelvic locations. DLP: Dose Length Product; GB: Gall Bladder; OAL: Other abdominal locations; RP: Retroperitoneum.

DLP outcomes values by age, sex and location are depicted in Fig. 1 and DLPs with multivariable adjustment for age, sex, anatomical location, number of helical acquisitions and inclusion center are presented in Table 1. The mean DLP was significantly higher for men than for women (*p* = 0.0005). It was also significantly different according to the targeted organ location (*p*<0.0001), higher for abdominopelvic procedures than for thoracic procedures. A trend towards higher dose for patients older than 60 years old was observed, although the difference was not statistically significant (*p* = 0.13).

**Table 1:** Influence of age, sex, location and number of helical acquisitions on total dose length product (DLP) for biopsies, punctures and drainages performed under CT guidance.

		Number (%)	Average DLP estimated in multivariable*	p-value
Age (years)	*x* < 60 yo	528 (30.9)	672.4 [579 - 780.9]	*0.1376*
	60 ≤ *x* < 71 yo	536 (31.4)	715 [615.4 - 830.7]	
	*x* ≥ 71 yo	645 (37.7)	715.3 [616.9 - 829.3]	
Sex	Women	664 (38.9)	665.4 [573.9 - 771.4]	*0.0005*
	Men	1045 (61.1)	737.7 [637.7 - 853.5]	
Location	Lung	760 (44.5)	466.2 [404.2 - 537.8]	*p <0.0001*
	Kidney	275 (16.1)	698.7 [598.4 - 815.9]	
	Retroperitoneum	141 (8.3)	762.5 [645.2 - 901.2]	
	Pelvis	102 (6)	710.3 [593.8 - 849.5]	
	Liver	96 (5.6)	723.4 [601.8 - 869.6]	
	Pancreas	39 (2.3)	875.2 [695.8 - 1100.9]	
	Gall bladder	28 (1.6)	800.2 [603.5 - 1061.1]	
	Adrenal	26 (1.5)	643 [495.2 - 835.1]	
	Other abdominal locations	242 (14.2)	707.5 [604.1 - 828.6]	

The “Other abdominal locations” item includes a very small number of procedures (*N*<20) of variable anatomic locations (mesenteric, parietal, intestinal, perivascular …).

The dose-length product means are presented with their 95% confidence interval [95% CI] and their corresponding p-values after exponential transformation of the linear regression estimates.

* Multivariable analyses were adjusted according to age, sex, anatomical location, number of helical acquisitions and inclusion center.

### 3.3 Imaging data

**Fig. 2:**
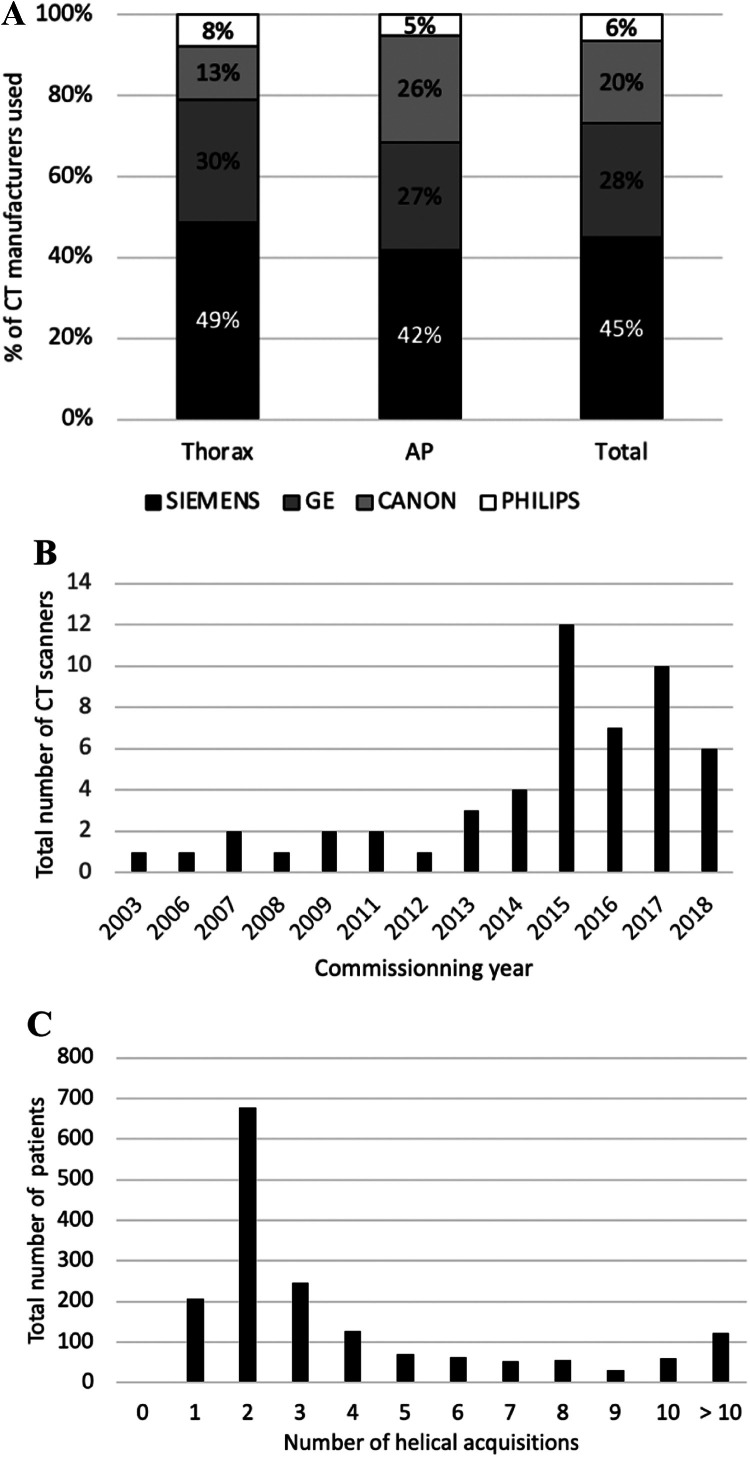
Technical aspect of the procedure: A. Proportion (%) of CT manufacturers used for all procedures, thoracic or abdomen pelvic. B. Distribution of commissioning year of the 52 CT systems used. C. Distribution of the total number of helical acquisitions for the 1709 patients.

Regarding the CT-scanner used, 767 of the 1709 procedures (45%) were performed using a Siemens CT system, 485 procedures (28.4%) using a GE system, 34 (20.3%) on a Canon CT system and 110 (6.4%) on a Philips CT system. On average, the commissioning year of the CT system used was 2014 ± 3.5 years (range 2003–2018). The median number of helical acquisitions was 2 (range 0–73) (Fig. 2).

## 4 Discussion

Our results showed that the doses delivered to the patients depend on gender and targeted organ location during CT guided biopsies, punctures and drainages for thoracic and abdominopelvic locations.

Biopsies are often separated from punctures/drainages in the literature such as thoracic or abdominopelvic locations. We gathered all biopsies, punctures and drainages because of the similarities in the technique (approach particularly). Also, a previous study showed doses delivered to the patients were equivalent for thoracic and abdominopelvic locations [9]. Their DLPs were in-between those of drainages and biopsies, in accordance with the literature [2,4].

In terms of dose delivered, our results are in accordance with the literature. Leng et al. [1] found a mean DLP of 909 mGy.cm-1 for biopsies, and Kloecklner et al. [2] have published similar dose levels between 545 mGy.cm-1 for pleural drainage and 889 mGy.cm-1 for retroperitoneal biopsy.

We showed that the total DLP was not dependent of the patient's age but was significantly different depending on the patient's sex. This may be explained by the different morphologies [3,10,11]. This result is in accordance with previous studies assessing the impact of the patient's height and weight on the dose received [3,10].

Our study also showed that the total DLP was significantly different depending on the organ involved. Indeed, it was lower for the lung than for abdominal organs, which confirmed previous study results [9]. This may be explained by a more rectilinear path to the target associated with a higher contrast between the nodule and the lung, requiring fewer iterative controls, thus generating a lower dose. The abdomen and pelvis are less contrasting and more attenuating locations thus requiring a higher dose. In addition, they may require more controls due to paths being often oblique and the need to avoid organs at risk. Kloecklner et al. also found different DLP depending on the biopsy location [2]. Doses were higher for retroperitoneal biopsies delivered higher than other locations. In accordance with our results, doses were lower for thoracic than abdominal biopsies [4].

Our study shows for the first time the patient-dependent factors also impact the dose delivered to the patients. Indeed, previous literature described the role of external factors such as the interventional radiologist practice and CT parameters in the delivered dose. We now show the importance of demographics factors such as sex and targeted organ location in the dose delivered during interventional procedures adjusted to the number of helical acquisitions.

Our study has some limitations. The main limitation is the absence of BMI record; indeed, it would seem interesting to carry out a systematic collection of BMI in future studies. Other patient data may also have been interesting to investigate. Further studies should for example study the impact of major patient comorbidities on the dose delivered to the patient. This may allow increasing more and more personalization of the dose delivered. Also, the use of the guidance system that could reflect the complexity of the puncture were not collected in this study. Abdominal collection was sometimes not precise enough and classified as “other abdominal locations”. Finally another limitation was the lack of information about kV, mAs, CTDI vol and acquisition length for each CT acquisition which would have allowed to assess a CT system effect on the dose.

## 5 Conclusion

This study demonstrated that the doses delivered to the patient for biopsy, puncture and drainage performed under CT guidance in thoracic and abdominopelvic locations, were gender and location dependent. There seems to be an interest in proposing finer dose reference levels according to the patient's morphology and anatomical location of the procedure.

## Funding

This research did not receive any specific grant from funding agencies in the public, commercial or not-for-profit sectors.

## CRediT authorship contribution statement

**Laure Berny:** Conceptualization, Data curation, Formal analysis, Investigation, Validation, Writing – original draft, Writing – review & editing. **Joël Greffier:** Conceptualization, Formal analysis, Formal analysis, Funding acquisition, Investigation, Methodology, Resources, Validation, Writing – review & editing. **Chris Serrand:** Formal analysis, Methodology, Software, Validation. **Djamel Dabli:** Data curation, Validation, Writing – review & editing. **Fabien De Oliveira:** Data curation, Validation. **Hélène de Forges:** Methodology, Validation, Writing – original draft. **Jean-Paul Beregi:** Conceptualization, Project administration, Resources, Supervision, Validation, Visualization, Writing – review & editing. **Julien Frandon:** Conceptualization, Formal analysis, Funding acquisition, Investigation, Methodology, Project administration, Resources, Supervision, Validation, Visualization, Writing – original draft, Writing – review & editing.

## Declaration of Competing Interest

The authors declare that they have no known competing financial interests or personal relationships that could have appeared to influence the work reported in this paper.
